# Volatile Organic Compound (VOC) Contamination in Hotel Rooms: A Pilot Study to Understand Sources and Health Risks

**DOI:** 10.3390/ijerph21111464

**Published:** 2024-11-02

**Authors:** Adam Nored, Xianqiang Fu, Rui Qi, Namuun Batbaatar, Chunrong Jia

**Affiliations:** 1 School of Public Health, University of Memphis, Memphis, TN 38152, USAxfu@memphis.edu (X.F.); namuun.b@memphis.edu (N.B.); 2 Kemmons Wilson School of Hospitality and Resort Management, University of Memphis, Memphis, TN 38152, USA; rqi1@memphis.edu

**Keywords:** volatile organic compounds, hotel, indoor, cancer risk, passive sampling

## Abstract

The COVID-19 pandemic drove the use of cleaning products, causing organic solvent contamination in hospitality environments. This pilot study investigated the presence and concentrations of volatile organic compounds (VOCs) in selected hotels in four different US cities with varying star ratings at the end of the pandemic period. Targeting 139 VOCs, 57 were detected across eight groups: alcohols, halocarbons, aromatics, alkanes, terpenes, carbonyls, ethers, and esters, in the indoor air. Alcohols were the most prevalent, especially in lower-rated hotels, suggesting higher use of cleaning supplies. Elevated levels of aromatics were detected in hotels rated under three stars, with a notable disparity compared to higher-rated hotels. Additionally, alkanes and terpenes such as n-tetradecane and d-limonene were consistently detected. Health risk assessment showed concentrations of all VOCs remained below their health criteria for customers. The cumulative cancer risk was 2.25 × 10^−6^ for hotel workers from chronic occupational exposure to eight carcinogenic VOCs, representing 1/3 of the lifetime risk from these chemicals in the ambient air. Cancer risks from individual VOCs ranged from 0.001 × 10^−6^ to 1.07 × 10^−6^, with chloroform accounting for nearly half of the cumulative risk. The findings underscore the need for careful selection and use of furnishings and cleaning supplies and for effective indoor air pollution control and management in hotel indoor environments.

## 1. Introduction

Indoor air quality (IAQ) emerges as a strategic asset for the hospitality sector in a post-pandemic context [1], impacting various stakeholders within the hotel industry. According to the latest data from Smith Travel Research in April 2023, the hotel occupancy rate has rebounded to approximately 95% of 2019 levels and continues to grow, both in the US and globally [2]. IAQ significantly affects hotel guests’ sleep quality satisfaction [3] and loyalty [4]. Notably, among various indoor environmental issues, IAQ complaints exert the most substantial influence on hotel online ratings [5]. The COVID-19 pandemic has further increased travelers’ demand for safety and health measures in hotels, with a particular emphasis on IAQ [6]. Moreover, the pandemic has intensified concerns for the safety and health of hotel workers in the post-pandemic work environment, especially housekeepers who face heightened exposure to increased indoor environmental hazards due to enhanced cleaning protocols [7,8]. Therefore, monitoring and managing IAQ is crucial for employee recruitment and retention, given the ongoing labor shortage crisis in the US hotel industry. Furthermore, improving hotel IAQ aligns with corporate social responsibility (CSR) principles by emphasizing environmental safety, which can be leveraged in green marketing to attract and retain green customers and investors. This aspect is particularly significant for hotels as the environment is a fundamental component of their product [9]. From the competitors’ standpoint, hotels face more IAQ challenges compared to Airbnb, considering their distinguished differences in locations and building structures [10,11]. Thus, enhancing hotel IAQ is crucial in influencing travelers’ choices between hotels and Airbnb.

Volatile organic compound (VOC) contamination is an outstanding IAQ issue in hotels. Hotel rooms, like residences, contain multiple sources that emit VOCs. Building materials, furnishings, and furniture are known to emit hundreds of VOCs. Carpets, a common feature in hotels, act as a primary source of VOCs in indoor environments [12,13]. Wall vinyl and furniture, particularly those made from composite wood, can off-gas formaldehyde and other VOCs [14]. An emission study showed that a footstool and a bedside table could elevate aromatic concentrations by 40−850 µg/m^3^ [15]. In real homes, new furniture materials could increase formaldehyde concentrations by 15–135 µg/m^3^ and aromatic concentrations by up to 9000 µg/m^3^ [16]. Wood-based future could cause indoor formaldehyde concentrations of up to 90 µg/m^3^ even after 7 years [17]. Human activities and behaviors are non-negligible indoor pollution sources. Smoking was historically a significant contributor to poor IAQ in hospitality settings [18,19]. Hotels use a large amount of cleaning agents to clean in between every guest. As a result, hotel indoor air may contain elevated concentrations of VOCs, causing high exposures among guests and workers. Studies have linked high VOC exposure to many adverse health effects, such as respiratory and sensory irritation, as well as some cancers [20]. The cleaning protocols that were implemented during the COVID-19 pandemic may continue for a long period, and thus, there is an essential need to understand the VOC contamination in hotels and other hospitality venues in the post-pandemic era.

Despite all these concerns and motivations, our extensive literature review focused on IAQ in hotels has yielded limited studies, most of them pre-pandemic. One pandemic-era study brought up the hardships of studies conducted in hotels due to issues in ventilation and airflow, even in high-quality hotels [21]. Their study mainly focused on CO_2_ levels when discussing this subject, but the same principle applies to VOCs in rooms not dispersing due to ventilation issues. Another study of pandemic-era IAQ concerns was focused on the different areas of the hotels to determine the hot spots for particulate matter and VOCs [1]. This case study focused on the challenges of keeping contaminant levels down throughout a hotel and noted that areas that required heavier cleaning had peak VOC levels. A pre-pandemic IAQ study involving four hotels reported that outside sources and indoor cleaning agents were the main contributors to indoor VOCs [22].

We conducted this convenience sampling study to obtain an understanding of organic air contaminants in hotels at the end of the COVID-19 pandemic. Specifically, we aimed to identify the species and concentrations of airborne VOCs by scanning for a wide range of target compounds, explore their sources, and estimate the potential health risks for hotel customers and workers. We expect the findings will guide effective sampling designs for similar hotel IAQ investigations, inform work practices to reduce indoor air pollution, and provide bases for measures of “green” hotels.

## 2. Materials and Methods

### 2.1. Hotel Selection and Sample Collection

This study adopted a convenience sampling approach for the collection of VOCs. Sample acquisition was initiated when a member of the research team was scheduled for a hotel stay. This study ended up with four hotels of varying star levels in four different US cities.

Hotel 1: This hotel was a 2.5-star extended-stay hotel in Memphis, TN, USA. The hotel room was on the 1st floor and had a small kitchenette included. Duplicate passive samples were collected from 2−5 August 2022. In addition, duplicate 20 min active samples were collected on 2, 3, and 4 August, respectively. This sampling event collected 8 samples plus 2 blanks.

Hotel 2: This hotel was a 4-star luxury hotel in Pittsburgh, PA, USA. The hotel room was on the 14th floor and had a traditional layout with no kitchenette. Two tubes were placed at two different spots within the main room and another sampler was in the bathroom from 21−25 August 2022. This sampling event resulted in the collection of 3 samples plus 1 blank.

Hotel 3: This hotel was a 3-star hotel in New York City, NY, USA. The hotel room was on the 14th floor and close to LaGuardia airport. One passive sampler was placed at the center of the room from 7−11 October 2022. This sampling event resulted in the collection of 1 sample plus 1 blank.

Hotel 4: This hotel was a 2-star hotel in Branson, MO, USA. The hotel room was on the 2nd floor of a 2-story hotel building. Duplicate passive samples were collected from 7−10 October 2022. The sampling event collected 2 samples plus 1 blank.

### 2.2. Indoor VOC Sampling Methods

Airborne VOCs were sampled using stainless-steel thermal desorption (TD) tubes packed with Tenax TA (Cat. No.: C1-AXXX-5003, Markes International, Llantrisant, UK). VOCs could be collected into the tube using passive or active sampling approaches, as used in our previous study [23]. In passive sampling, the tube was placed and fitted with a diffusion cap (Cat. No.: C-DF010, Markes International, Llantrisant, UK) for 3 or 4 days, depending on the stay time. The passive sampling rate ranged from 0.13−0.43 mL/min for specific VOCs [24], and the average sampling volumes were 1.22 L and 1.62 L for 3-day and 4-day sampling durations, respectively. In active sampling, a 4 L air sample was collected at a flow rate of 200 mL/min for 20 min with a tube connected to a pump (SKC AirChek TOUCH, SKC Inc., Eighty Four, PA, USA). A passive sample provides an estimate of sub-chronic exposure, and an active sample provides a snapshot of indoor VOC contamination. During the deployment, the passive and active samplers were positioned in a similar way. A tube was fixed on a stand and placed in the breathing zone (1.5−2.0 m from the ground). The stand was placed on a desktop, at least 0.5 m away from the wall.

### 2.3. Laboratory Analysis of VOC Samples

All the TD tube samples, including passive, active, and blank samples, were analyzed in the laboratory following a well-established procedure [24]. In brief, a tube was thermally desorbed in an automated TD system (Model: UltraA/Unity2, Markes International, Llantrisant, UK). The desorbed VOCs were then separated and analyzed on a coupled gas chromatography/mass spectrometry (GC/MS) system (Agilent 7890A/5975C, Agilent Technology Inc., Santa Clara, CA, USA) operated in a scan mode. The TD-GC/MS operating parameters are summarized in Table S1. The spectra of analytes were analyzed for 139 target compounds (Table S2) in ChemStation (Version B.07.04.2260, Agilent Technology Inc., Santa Clara, CA, USA). The method detection limits were 0.0046–0.63 ng, or 0.0232–2.99 µg/m^3^, for 4 L active samples and 0.008–0.043 µg/m^3^ for 3-day passive samples (Table S2). ΣVOCs was defined as the sum concentration of all the detected VOC concentrations in any sample. For undetected compounds, we used half of the method detection limit (MDL).

### 2.4. Quality Assurance and Quality Control (QA/QC)

The TD tubes were cleaned in a tube conditioner (model: TC-20, Markes International, Llantrisant, UK) at 335 °C with 60 mL/min of ultra-high purity grade helium for two hours before use. They were then sealed with storage caps and stored inside a VOC-free refrigerator until use. Field blanks were collected on the starting day of every sample collection. The sampling flows for active sampling were pre-calibrated using a primary flow meter (Defender 220 Medium Flow, Mesa Laboratories, Inc., Butler, NJ, USA) and were rechecked right after the field deployment. The TD system was equipped with an internal standard (IS) module that added a controlled amount of IS gas to each sample. The MS calibration and mass calculations were based on the IS gas to account for any GC/MS variations throughout the different sample runs. The continuing calibration verification was performed by analyzing a known standard at the mid-range of the calibration curve, and recovery within ±30% of the nominal mass was considered acceptable. Two early-eluting alcohols were not detected for samples collected in Hotel 3 and Hotel 4 due to a retention time shift, and their concentrations were treated as missing.

### 2.5. Health Risk Assessment

Health risks were evaluated for customers and hotel workers. For customers, the short-term non-cancer risks were evaluated by comparing the indoor concentration of a chemical with its intermediate minimum risk level (MRL), which was designed for intermediate exposure duration from 1 to 14 days [25]. The hazard quotient (HQ) of a chemical was calculated as the quotient of the concentration divided by its MRL, and the cumulative non-cancer risk was indicated by the hazard index (HI), calculated as the sum of individual HQs. Cancer risks were not evaluated for customers as cancers are considered health consequences due to chronic or lifetime exposures. For hotel workers, the occupational exposure concentration C_indoor-air-adjusted_ of a chemical was calculated as Equation (1):

(1) $$C_{\text{indoor-air-adjusted}}\;=C_{\text{indoor-air}}\;\times \;\frac{\mathrm{ET}\;\times \;\mathrm{EF}\;\times \;\mathrm{ED}\;}{\mathrm{AT}}$$

where C_indoor-air_ = the measured indoor concentration (µg/m^3^) of the chemical of interest; ET = exposure time (h/day) is 8 h/day for workers; EF = exposure frequency is assumed to be 219 days a year (5 work days/week minus 10 federal holidays, 4 weeks of vacation, and an additional 12 personal days); ED = exposure duration is the occupational tenure. It is assumed to be 10.4 years for workers in the service sector if a person can live over 65 years old, according to Table 16-106 of EPA’s Exposure Factors Handbook [26]; AT = averaging time is equivalent to the lifetime of 70 years (613,200 h). As the exposure occurs in the workplace, non-cancer risks should be evaluated by comparing with the occupational standards or guidelines, e.g., permissible exposure levels (PELs) or threshold limit values (TLVs). PELs and TLVs for VOCs are typically very high, e.g., often greater than 1000 µg/m^3^. C_indoor-air-adjusted_ of the detected VOCs did not exceed these thresholds, and thus, there is no need to conduct the assessment for non-cancer risks for hotel workers. We were more interested in whether there were significant cancer risks if workers had chronic exposures to the carcinogenic VOCs. The cancer risk for a carcinogenic VOC was calculated as Equation (2):

Risk = C_indoor-air-adjusted_ × IUR (2)

where IUR = inhalation unit risk (per µg/m^3^), taken from EPA’s Integrated Risk Information System [27]. The median Cindoor-air-adjusted of each compound was used for calculations. The cumulative cancer risk was defined as the total risk of all the carcinogenic VOCs detected in hotel rooms.

## 3. Results and Discussion

### 3.1. Presence and Concentrations of Airborne VOCs in Hotel Rooms

Out of the 139 target compounds, 57 were detected in this study. In our examination of VOCs within four hotels of varying star levels, we categorized the VOCs into eight groups: alcohols, halocarbons, aromatics, alkanes, terpenes, carbonyls, ethers, and esters (Table 1). Alcohols emerged as the predominant group, with concentration in Hotel 1 reaching 4872 µg/m^3^, constituting 90% of ΣVOCs, followed by Hotel 2 with 838 µg/m^3^ (95%). Isopropyl was notably high in Hotel 1, contributing significantly to the overall alcohol group.

Hotels 1 and 4, both rated under 3 stars, exhibited elevated levels of aromatics, with concentrations at 37.1 µg/m^3^ and 41.0 µg/m^3^, respectively. Lower concentrations of aromatics were found at 13.8 µg/m^3^ in the 3-star Hotel 3 and at 4.38 µg/m^3^ in the 4-star Hotel 2. Alkanes and terpenes were consistently found across all hotels, with n-tetradecane and d-limonene being the predominant VOCs within their respective groups. Halocarbons were relatively low in all hotels, with chloroform as the predominant compound, peaking at 12.7 µg/m^3^ in Hotel 1. We found no detectable levels of 2,5-dimethylfuran, a tobacco smoke marker [28], suggesting that all hotels studied adhered to the smoking-free policy.

Spatial variation of indoor VOC concentrations was examined in Hotel 1 and Hotel 2 (Table S3). In Hotel 1, one sampler was placed near the kitchenette and the other near the bed. The concentrations measured by these two samplers were similar, with most percent differences below 10%. VOC concentrations within the small hotel room could be considered uniform. In Hotel 2, one sampler was placed in the bedroom, but another was in the bathroom. VOC concentrations, in general, were higher in the bathroom. The elevated concentrations were particularly obvious for cleaning-related chemicals like alcohols and tap water disinfectants like chloroform. In addition, activities such as bathing or showering might also be factors, as evidenced by the increased levels of terpenes, which are ingredients of personal care products.

### 3.2. Potential Sources of VOCs in Hotels

VOCs in hotels originate from diverse sources, including building materials, furnishings, and human activities. Carpets, a common feature in hotels, act as a primary source of VOCs in indoor environments [12,13]. Wall vinyl and furniture, particularly those made from composite wood, can off-gas formaldehyde and other VOCs [14]. Smoking, historically a significant contributor to poor indoor air quality in hospitality settings [18,19], has been largely eliminated in many jurisdictions. Recent studies in the hospitality industry have shown that regulations against smoking on premises have significantly improved indoor air quality compared to previous field studies [29].

Table 2 presents potential sources of the predominant VOCs detected in hotel rooms. While some VOCs might originate from external sources, others were directly related to cleaning, with few alternative sources that would be seen in a hotel setting. Alcohols such as ethyl alcohol, isopropyl alcohol, and 2-butoxyethanol are commonly found in sanitizers, disinfectants, antibacterial sprays, all-purpose cleaners, and deodorizers [30]. These VOCs typically have the highest concentrations, so elevated levels were anticipated compared to other compounds. For example, the levels of these alcohols in a lower-rated hotel (Hotel 1) were significantly higher than those found in a higher-rated hotel (Hotel 2). This suggests that a considerably larger amount of cleaning supplies is used daily at the low-rated hotels.

Concentrations of certain alkanes, such as n-tetradecane and n-pentadecane, were notably higher in Hotel 1 compared to other hotels, as indicated in Table 1. While many alkane emissions can be linked to outside sources, heavy alkanes such as n-tetradecane and n-pentadecane are mostly indoor sourced. They are components of building materials and pest control products.

The high concentration of 2-ethyl-1-hexanol (101 µg/m^3^) in Hotel 1 caught our attention. This chemical has many uses, such as flooring adhesives and plastics [33]. It may also be emitted from the degradation of building materials due to water damage or when some materials are left in stagnant water [34]. There was observed standing water in the parking lot of Hotel 1 due to several days of heavy rains, leading to a possible cause. Fragrance chemicals, including 2-ethyl-1-hexanol, have similarly been identified in other environments, like nail salons, where they are associated with emissions from cleaning and personal care products [49]. This further explains the overlap of VOC sources among those commercial settings.

Overall, the levels of VOCs appeared to be influenced by the hotels’ rating and quality, as indicated by their stars. The difference in rating often correlates with the quality of materials used for the hotels’ furnishings. Lower-rated hotels exhibited higher concentrations of VOCs, such as ethyl acetate, m,p-xylene, and styrene, which are found in products used in the creation and finishes of furniture and room furnishings [38,39,50]. The types of cleaning supplies might have played a role, too. Higher-rated hotels are more likely to use higher-quality and less harsh cleaning agents. Additionally, there has been a growing trend among higher-rated hotels towards “going green,” which includes switching to environmentally friendly cleaning agents. This shift could contribute to the lower concentrations of VOCs observed in these more upscale hotels.

### 3.3. Health Risks from Indoor VOC Exposure

For hotel customers, their short-term VOC exposures in hotels were below the corresponding MRLs (Table 1). Certain compounds, such as 2-butoxyethanol and 2-ethyl-1-hexanol, while below their respective MRLs, were among the higher concentrations measured. Benzene had a median concentration representing 11% of its established MRL. In Hotel 4, benzene had a significant spike at the concentration of 27.9 µg/m^3^, which was close to its MRL of 29 µg/m^3^. The HI of multiple VOC exposure was 0.13, suggesting a small cumulative non-cancer risk (Table S5). According to the currently available MRLs, VOC levels in hotel rooms do not pose short-term health risks to the guests. However, a complete evaluation of the risks still needs more research, with MRLs unavailable for many VOCs and potential interactions of complex VOC mixtures.

Table 3 presents the cancer risks of hotel workers from VOC exposures during their work time. Among the fifty-seven detected compounds, eight compounds were classified as carcinogens. The risks of occupational exposure to these individual chemicals ranged from 0.001 × 10^−6^ to 1.07 × 10^−6^, and their cumulative cancer risk was 2.25 × 10^−6^. Chloroform presented the most significant individual cancer risk of 1.07 × 10^−6^, accounting for nearly half (47%) of the cumulative risk. Benzene followed, contributing 33% to the cumulative risk with an individual risk value of 0.75 × 10^−6^. Naphthalene, 1,2-dichloroethane, 1,4-dichlorobenzene, and carbon tetrachloride had small risk contributions ranging between 1% and 10%. Tetrachloroethylene, a phased-out dry-cleaning solvent, had a negligible risk. For the purpose of comparison, we took national average cancer risks from ambient air toxics estimated by the latest national-scale air toxics program [51], which could be regarded as the baseline risk level. The indoor VOC profiles are quite different from outdoor VOC profiles, as observed in numerous indoor and outdoor VOC studies. Accordingly, the cancer risks of individual chemicals were quite different between indoor and outdoor environments. The cumulative risk in hotels was about 1/3 of the baseline risk (Table 3), despite that the occupational exposure duration in hotels represents only 3% of the lifetime. This fact suggests that occupational VOC exposure contributed to a sizable fraction of the lifetime cancer risks among hotel workers.

### 3.4. Comparison with Previous Studies

Indoor VOCs have been understudied for hotels compared to other indoor environments in the US. In the only previous US study, the cancer risk was 4.5 × 10^−6^ among hotel workers, if applying the same set of VOCs and exposure factors as our study [52]. This risk was on the same order of magnitude as the median risk of 2.25 × 10^−6^ in our study. A strict comparison between the two studies was not possible due to small sample sizes, lack of original data, and many uncertainties in indoor VOC measurements. Another relevant investigation evaluated indoor air quality across 13 new hotels in southern China [53]. This study reported that aromatic compounds, e.g., toluene, m,p-xylenes, ethylbenzene, and methylene chloride, were predominant in guest rooms, comprising 62% of total VOC concentrations. The authors speculated that indoor air pollutants in hotels might originate from various sources, including cleaning agents, outdoor pollution, and materials used in room decor. Our study adapted its focus considering the COVID-19 pandemic by including alcohols and fragranced chemicals in our list of targeted VOCs, reflecting a shift towards increased use of these substances in hotel cleaning protocols. This adjustment aimed to address heightened sanitation practices and odor-masking concerns, providing a more nuanced view of IAQ in hotel settings.

### 3.5. Implications for Indoor VOC Sampling Strategies

Our field sample collection employed several sampling strategies that could inform future indoor VOC sampling in hotel rooms. As presented above, the similarities in VOC levels at two locations within a hotel room (Table S3) suggest that a single location could capture representative indoor VOC concentrations. The additional sample in the bathroom may help identify sources of VOCs relating to tap water, cleaning products, and personal care products (Table S3).

**Figure 1:**
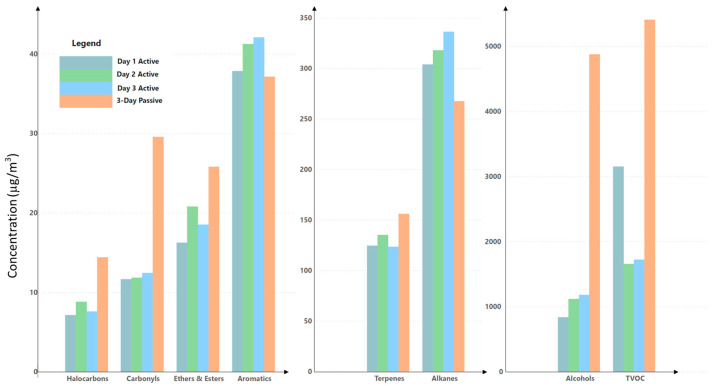
Comparison of VOCs by sampling method (active vs. passive) over 3 days.

We also used passive and active sampling methods to evaluate sub-chronic and acute exposures. The passive sampling lasted 3 or 4 days, providing an estimate of sub-chronic exposure. The active sampling lasted only 20 min and measured a snapshot of contamination. Figure 1 presents a comparison between the two methods, and Table S4 has detailed comparison data for individual compounds. The two methods yielded generally comparable results. Alcohols had markedly higher readings from passive sampling, suggesting a more pronounced accumulation over several days. For instance, passive sampling recorded an average of 4872 µg/m^3^ for alcohols across three days, while three 20 min active samples yielded concentrations ranging from 835 to 1179 µg/m^3^, potentially missing concentration spikes. The discrepancy in readings between passive and active sampling might be attributed to the dynamics of indoor airflows and the temporal release patterns of VOCs from various sources. Halocarbons, ethers, esters, terpenes, and carbonyls followed similar patterns to alcohols, with the exception of terpenes and aromatics, which showed less variation between sampling methods. This could suggest a more uniform release rate of terpenes and aromatics into the indoor environment, or it could reflect their chemical properties affecting their uptake rate by the sampling media. These results suggest that passive sampling is a feasible method for assessing short-term to long-term indoor VOC exposures. Active sampling remains a convenient tool for obtaining an instant assessment of IAQ.

### 3.6. Implications for Hotel IAQ Management

The COVID-19 pandemic has reshaped the way travelers perceive their health and safety during their hotel stay. For instance, a 2020 survey showed that 77% of US consumers consider hotel IAQ in their decision making, and 52% are willing to pay a premium for rooms with better IAQ [54]. This shift in demand prompted major hotel chains to invest in cleaning and hygiene protocols as a new competitive edge [55]. As a result, 79% of hospitality operators have revised their cleaning protocols for hotel rooms due to the pandemic [56]. These practices are likely to continue post-pandemic, supported by the projected growth of the global hotel cleaning services market at a 6.7% annual rate from 2024 to 2032 [57].

The findings of this study suggest indoor organic chemical contamination has not posed a significant health risk to hotel guests. This good news, however, does not downplay the importance for hotels to continue investing in monitoring and improving IAQ post-pandemic to enhance guest health, thereby building their trust and satisfaction. As opening windows may not always be feasible, hotels can implement IAQ monitors, update ventilation systems, and place air purifiers in critical areas such as rooms near kitchens and laundry rooms. For instance, Pure Wellness partners with over 200 US hotels to provide Pure Rooms equipped with medical-grade air purifiers, targeting allergy-sensitive or wellness travelers willing to pay extra for cleaner air [58]. However, although Pure Rooms cater to more luxury segments, our findings indicate that lower-star hotels may face greater IAQ health risks. Therefore, it is crucial for these hotels to adopt cost-effective methods to improve IAQ, such as using natural ventilation, IAQ sensors, and green cleaning products.

Additionally, the findings of this study underscore the critical need to protect hotel employees, particularly housekeepers, from the health risks associated with frequent exposure to large quantities of cleaning solvents, an often overlooked workplace hazard. Current protection measures focus on providing personal security devices and limiting the amount of floor space cleaned daily to reduce musculoskeletal injuries. Our findings emphasize the necessity for hotels to address IAQ issues for housekeepers, which could include providing safer cleaning chemicals, improving ventilation during cleaning, supplying personal protective equipment, and offering educational training [59].

Lastly, while upgrading ventilation systems can be costly, it is becoming increasingly financially beneficial for hotel owners and operators focused on returns. Hospitality investors are aiming to build more sustainable hotel assets, and environmental, social, and governance (ESG) factors—especially environmental considerations—are reshaping their investment decisions and affecting hotel asset valuations. Banks are more willing to lend to green hotel assets and offer lower borrowing costs [60], and insurance companies prefer to insure properties that mitigate environmental risks. For example, lodging real estate investment trusts (REITs) are including IAQ performance as part of their environmental initiatives in hotel development and renovation projects [61]. Additionally, the American Hotel & Lodging Association is developing the Green Key Global sustainability certification, which requires hotels to assess and audit their environmental performance, including IAQ. In summary, changing investor and insurer attitudes, along with new sustainability certifications, provide financial incentives for hoteliers to invest in IAQ. This not only addresses health concerns but also offers a strong return on investment by enhancing asset valuation and improving access to financial capital. Moreover, hotels can incorporate their IAQ initiatives into CSR marketing and ESG reporting, effectively communicating these efforts to stakeholders including consumers, equity investors, and lenders.

### 3.7. Study Limitations

While this study provides valuable insights into the presence and concentrations of VOCs in hotel environments, it presents several limitations. The analysis was confined to just four hotel rooms, which could not represent the wide diversity of hotel operations and management practices. Future research should expand to include a larger sample of hotels, ideally capturing variations across different geographical locations and hotel categories. We also suggest longitudinal measurements to track the trends in indoor VOC contamination in hotels in the post-pandemic era. Future studies may use a continuous monitor, e.g., a photoionization detector [62], to capture the temporal variation in VOC concentrations due to the dynamics of hotel operations. Outdoor VOC concentrations were not measured as permission was not granted. Our study focused primarily on specific types of VOCs without considering other IAQ factors that could influence or be influenced by VOC concentrations. Parameters such as temperature, humidity, CO_2_ levels, ventilation rates, and even subjective measures like odor assessments should be incorporated into future studies to provide a more comprehensive understanding of the IAQ in hotels. A more detailed survey or questionnaire regarding hotel cleaning practices could also enhance understanding of the sources of VOCs. Information on the types of cleaning agents used, the frequency of cleaning, and the training of housekeeping staff could be particularly informative.

While we analyzed VOC levels in hotels, we did not examine how stakeholders perceive improvements in IAQ. Future research could assess whether consumers are willing to pay a premium for better IAQ. By applying the theory of planned behavior [63], researchers can explore how changing social norms toward sustainability influence consumer behavior in favoring enhanced IAQ practices. Additionally, understanding the barriers and opportunities from the perspectives of owners, operators, and investors is crucial. Using the resource-based view [64], future studies can investigate how IAQ improvements contribute to sustainable competitive advantage. To gain these insights, focus groups and surveys involving these stakeholders should be conducted. This approach addresses the other half of the equation—making a persuasive argument for behavioral changes—and can help develop strategies for sector-wide adoption of IAQ improvements. By addressing these limitations, future research can provide more robust data and insights, helping to improve IAQ management in hotels and enhance the health and safety of both guests and staff.

## 4. Conclusions

This study detected 57 VOCs in the indoor air of hotel rooms and revealed alcohols, probably related to cleaning activities, as the most prevalent VOCs. Elevated levels of VOCs were found in lower-rated hotels, warranting further studies of the emissions in these hotels, e.g., building materials and cleaning practices. Indoor VOC levels were below the short-term health guideline values, suggesting the unlikelihood of non-cancer symptoms or diseases among hotel guests during their stay. However, high concentrations of 2-butoxyethanol, 2-ethyl-1-hexanol, and benzene, sometimes close to the health criteria, may pose health risks. Occupational exposure to eight carcinogenic VOCs detected in this study had a cumulative cancer risk of 2.25 × 10^−6^, accounting for one-third of the baseline lifetime cancer risk of the same compounds. The findings underscore the critical need for regular monitoring of IAQ in hotels, particularly those with lower star ratings or those not adhering to green cleaning practices. By understanding and managing VOC levels, hotel management can significantly enhance the health and safety environment, aligning with both guest expectations and health regulations.

## Figures and Tables

**Table 1:** Major VOC groups and selected predominant VOC species detected in hotel rooms, measured in µg/m^3^.

VOCs	Hotel 1 (2.5-Star)	Hotel 2 (4-Star)	Hotel 3 (3-Star)	Hotel 4 (2-Star)	Median	MRLs
Alcohols	4872	838	115	96	477	
Ethyl alcohol	1597	615	n.a.	n.a.	1106	n.a.
Isopropyl alcohol	3046	210	n.a.	n.a.	1628	n.a.
2-Butoxyethanol	122	6.75	52.9	3.71	29.8	2900
2-Ethyl-1-hexanol	101	4.14	59.0	84.0	71.5	n.a.
Halocarbons	14.4	3.70	0.35	0.45	2.07	
Chloroform	12.7	3.11	0.01 *	0.01 *	1.56	490
Aromatics	37.1	4.38	13.8	41.0	25.5	
1,4-Dichlorobenzene	11.3	0.03 *	0.24	0.22	0.23	12,000
Benzene	3.71	0.56	2.73	27.9	3.22	29
Toluene	5.30	0.88	2.32	5.08	3.70	7500
Ethylbenzene	0.95	0.27	0.65	0.71	0.68	22,000
Xylenes	4.89	1.20	2.80	3.50	3.15	8700
Styrene	1.87	0.25	2.83	0.35	1.11	21,000
Naphthalene	4.58	0.13	0.21	0.23	0.22	n.a.
Alkanes	267	7.03	90.9	15.8	53.3	
n-Tetradecane	179	0.65	6.05	4.86	5.46	n.a.
n-Pentadecane	69.5	0.73	1.87	1.66	1.76	n.a.
Terpenes	156	22.1	94.4	48.6	71.5	
d-Limonene	63.1	8.75	57.5	17.7	37.6	n.a.
β-Pinene	8.15	0.87	4.18	2.72	3.45	n.a.
α-Terpinene	1.52	2.73	14.6	0.07	2.13	n.a.
Linalool	40.0	2.86	0.12 *	7.60	5.23	n.a.
Menthol	13.2	2.55	9.87	15.5	11.5	n.a.
Terpineol	8.20	0.50	0.12 *	0.12 *	0.31	n.a.
Ethers and Esters	25.8	3.96	7.47	4.07	5.77	
Ethyl acetate	8.86	1.20	6.33	2.94	4.64	n.a.
Methyl salicylate	4.51	0.68	0.09 *	0.09 *	0.39	n.a.
Tetrahydrofuran	7.69	1.08	0.04 *	0.04 *	0.56	n.a.
Carbonyls	29.5	4.14	13.7	13.8	13.8	
Benzaldehyde	27.4	3.89	13.5	13.5	13.5	n.a.
ΣVOCs	5404	882	339	217	610	

* Values below the detection limit have been replaced with half of the method detection limit (MDL); MRLs—Minimal risk levels for hazardous substances by ATSDR; n.a.—not available.

**Table 2:** Potential sources of the predominant VOCs detected in hotel rooms.

VOCs	Possible Emissions Sources
Alcohols	
Ethyl alcohol	Sanitizer, disinfectant [30,31]
Isopropyl alcohol	Sanitizer, disinfectant, deoderizer [30,31]
2-Butoxyethanol	All-purpose cleaner [32], cleaner [30,31]
2-Ethyl-1-hexanol	Plastics, wood stain [33], water damage [34]
Halocarbons	
Chloroform	Tap water, bleach [35]
1,4-Dichlorobenzene	Deodorizer, air freshener [36]
Aromatics	
Benzene	Wood stain, adhesives [37], exhaust [38]
Toluene	Antibacterial spray [32], paint [30], exhaust [38]
Styrene	Wax, resins [39]
Naphthalene	Deodorizer [36], pesticide, grass killer [40]
Alkanes	
n-Tetradecane	Disinfectant [30], pest control, biocide [41]
n-Pentadecane	Pest control, biocide [41]
Terpenes	
d-Limonene	Air freshener [37], detergent, cleaner [30]
β-Pinene	Antibacterial cleaner [32], disinfectant [30]
α-Terpinene	Cleaner [31], toilet deodorizer [37]
Linalool	Air freshener [31], fragrance [42]
Menthol	Air freshener [31], moist wipes [43]
Terpineol	Cleaner, air freshener [37]
Ethers and Esters	
Ethyl acetate	Air freshener [30], nail polish [30], exhaust [44]
Methyl salicylate	Fragrance, soap [45]
Tetrahydrofuran	Exhaust [46,47]
Carbonyls	
Benzaldehyde	Vehicle exhaust [48], cosmetics [43]

**Table 3:** Cancer risks from VOC exposure among hotel workers.

VOCs	Median (µg/m^3^)	IUR ^1^ (×10^−6^)	Risk (×10^−6^)	Contrib ^2^ (%)	Nat’l Ave ^3^ (×10^−6^)
Benzene	3.22	7.8	0.75	33	1.91
Ethylbenzene	0.68	2.5	0.05	2	0.23
Naphthalene	0.22	34	0.22	10	0.82
Chloroform	1.56	23	1.07	47	n.a.
1,2-Dichloroethane	0.03	26	0.02	1	0.026
1,4-Dichlorobenzene	0.23	11	0.08	3	0.017
Carbon tetrachloride	0.38	6	0.07	3	3.02
Tetrachloroethylene	0.13	0.26	0.001	0.04	0.006
Cumulative ^4^	6.45		2.25	100	6.03

^1^ IUR—Inhalation unit risk, data obtained from EPA’s Integrated Risk Information System. ^2^ Contribution to the cumulative risk. ^3^ National average risk, estimated by the 2019 National-scale Air Toxics Assessment (NATA) program. ^4^ Cumulative cancer risk was defined as the sum of cancer risks from the eight carcinogenic VOCs detected in this study.

## Data Availability

Data are contained within the article and supplementary materials.

## Supplementary Materials

The following supporting information can be downloaded at: https://www.mdpi.com/article/10.3390/ijerph21111464/s1, Table S1: Condition of thermal desorption-gas chromatography/mass spectrometry (TD-GC/MS) analysis of volatile organic compounds (VOCs); Table S2: Performance of TD-GC/MS analysis of target VOCs; Table S3: Spatial variation of VOC concentrations (µg/m^3^) in two hotel rooms; Table S4: VOC concentrations (µg/m^3^) measured by passive and active sampling methods in a hotel room; Table S5. Potential Risk of Major VOC groups and selected predominant VOC species detected in hotel rooms, measured in µg/m^3^.
