# Supplementary material for: Volatile Organic Compound (VOC) Contamination in Hotel Rooms: A Pilot Study to Understand Sources and Health Risks
Source: Int J Environ Res Public Health. 2024 Nov 2;21(11):1464. doi: 10.3390/ijerph21111464 (PMC11594154; doi:10.3390/ijerph21111464)
Supplement: Supplementary file 1 [file ijerph-21-01464-s001.zip › ijerph-3263711-supplementary.pdf]

International Journal of
*Environmental Research and Public Health*

MDPI

*Communication*

# Volatile organic compound (VOC) contamination in hotel rooms: A pilot study to understand sources and health risks

## Supplementary Materials

**Table S1.** Condition of thermal desorption-gas chromatography/mass spectrometry (TD-GC/MS) analysis of volatile organic compounds (VOCs).

| Parameters | Conditions |
|---|---|
| **TD** | |
| Instrument | TD UtraA Autosampler coupled with Unity2 (Markes International) |
| Focusing trap | "Air Toxics Trap" (Part# U-T15ATA-2S) |
| Tube desorption | 260°C (5min) with 50mL/min tube flow (split flow: 20mL/min) |
| Trap desorption | 25°C to 280°C (3min) at a maximum heating rate with 20mL/min trap flow |
| Outlet split-flow | 8mL/min |
| Flow path | 140°C |
| **GC** | |
| Column | HP-5ms Ultra Inert (30m×250μm×0.25μm, Agilent Technology) |
| Carrier gas | Helium (Ultra High Purity 5.0 Grade, Part# HE UHP300, Airgas Inc.) |
| Colum flow | 1.2 mL/min, Constant Flow |
| **GC oven ramp** | |
| Initial temperature | 35 °C, hold 3min |
| Final temperature | 300 °C; start at 15 °C/min to 95 °C (hold 2min), then 15 °C/min to 140 °C, then 35°C/min to 220 °C, and then 40°C/min to 300 °C (hold 4min). |
| Total Run Time | 20min |
| **MS** | |
| Aux heater | 280 °C |
| Ion source | 230 °C |
| Electron energy | 70 volts |
| Ionization mode | Electron ionization (EI) |
| Mass Range | Scan Mode, 35-350 amu |

**Table S2.** Performance of TD-GC/MS analysis of target VOCs.

| # | Target VOCs | CAS | RT (min) | MDLs (ng) | MDL-3d (μg/m³) |
|---|---|---|---|---|---|
| 1 | 1,1-Dichloroethylene | 75-35-4 | 1.879 | 0.037 | 0.015 |
| 2 | Acrylonitrile | 107-13-1 | 1.907 | 0.055 | 0.019 |
| 3 | Dichloromethane | 75-09-2 | 1.943 | 0.172 | 0.088 |
| 4 | Carbon Disulfide | 75-15-0 | 1.958 | 0.271 | 0.096 |
| 5 | trans-1,2-Dichloroethylene | 107-13-1 | 2.074 | 0.055 | 0.019 |
| 6 | Methyl Tert Butyl Ether | 1634-04-4 | 2.100 | 0.052 | 0.043 |
| 7 | 1,1-Dichloroethane | 75-34-3 | 2.157 | 0.018 | 0.007 |
| 8 | Propionitrile | 107-12-0 | 2.168 | 0.024 | 0.009 |
| 9 | n-Hexane | 114-54-3 | 2.284 | 0.090 | 0.045 |
| 10 | Methacrylonitrile | 126-98-7 | 2.314 | 0.043 | 0.017 |
| 11 | cis-1,2-Dichloroethylene | 156-59-2 | 2.370 | 0.020 | 0.008 |
| 12 | Ethyl Acetate | 141-78-6 | 2.374 | 0.059 | 0.030 |
| 13 | Methyl Acrylate | 96-33-3 | 2.430 | 0.038 | 0.020 |
| 14 | 2,2-dichloropropane | 594-20-7 | 2.430 | 0.106 | 0.049 |
| 15 | Bromochloromethane | 74-97-5 | 2.449 | 0.005 | 0.003 |
| 16 | Chloroform | 67-66-3 | 2.456 | 0.026 | 0.013 |
| 17 | Tetrahydrofuran | 109-99-9 | 2.558 | 0.204 | 0.084 |
| 18 | 1,1,1-Trichloroethane | 71-55-6 | 2.749 | 0.047 | 0.020 |
| 19 | 1,2-Dichloroethane | 107-06-2 | 2.786 | 0.031 | 0.017 |
| 20 | 1,1-Dichloropropene | 563-58-6 | 2.873 | 0.027 | 0.012 |
| 21 | Benzene | 71-43-2 | 2.940 | 0.059 | 0.033 |
| 22 | Carbontetrachloride | 56-23-5 | 2.951 | 0.030 | 0.015 |
| 23 | 1,2-Dichloropropane | 78-87-5 | 3.742 | 0.040 | 0.019 |
| 24 | n-Heptane | 142-82-5 | 3.480 | 0.052 | 0.032 |
| 25 | Trichloroethylene | 79-01-6 | 3.491 | 0.029 | 0.014 |
| 26 | Dibromomethane | 74-95-3 | 3.517 | 0.033 | 0.014 |
| 27 | Bromodichloromethane | 75-27-4 | 3.600 | 0.025 | 0.011 |
| 28 | 2,5-Dimethylfuran | 625-86-5 | 3.630 | 0.036 | 0.017 |
| 29 | Methyl Methacrylate | 80-62-6 | 3.716 | 0.030 | 0.016 |
| 30 | trans-1,3-Dichloropropene | 10061-02-6 | 4.170 | 0.026 | 0.012 |
| 31 | cis-1,3-Dichloropropene | 10061-01-5 | 4.601 | 0.030 | 0.014 |
| 32 | Toluene | 108-88-3 | 4.612 | 0.039 | 0.021 |
| 33 | 1,1,2-Trichloroethane | 79-00-5 | 4.694 | 0.018 | 0.009 |
| 34 | 1,3-Dichloropropane | 142-28-9 | 4.927 | 0.021 | 0.010 |
| 35 | Ethyl methacrylate | 97-63-2 | 4.976 | 0.081 | 0.043 |
| 36 | Dibromochloromethane | 124-48-1 | 5.077 | 0.023 | 0.011 |
| 37 | n-Octane | 111-65-9 | 5.167 | 0.117 | 0.075 |
| 38 | 1,2-Dibromoethane | 106-93-4 | 5.260 | 0.027 | 0.022 |
| 39 | Tetrachloroethylene | 127-18-4 | 5.324 | 0.022 | 0.012 |

| # | Target VOCs | CAS | RT (min) | MDLs (ng) | MDL-3d (μg/m³) |
|---|---|---|---|---|---|
| 40 | Chlorobenzene | 108-90-7 | 5.916 | 0.045 | 0.022 |
| 41 | 1,1,1,2-Tetrachloroethane | 630-20-6 | 5.980 | 0.025 | 0.013 |
| 42 | Ethylbenzene | 100-41-4 | 6.160 | 0.033 | 0.016 |
| 43 | m,p-Xylene | 108-38-3/106-42-3 | 6.284 | 0.079 | 0.043 |
| 44 | Bromoform | 75-25-2 | 6.505 | 0.056 | 0.027 |
| 45 | Styrene | 100-42-5 | 6.599 | 0.057 | 0.023 |
| 46 | o-Xylene | 95-47-6 | 6.632 | 0.048 | 0.027 |
| 47 | n-Nonane | 111-84-2 | 6.722 | 0.047 | 0.033 |
| 48 | 1,1,2,2-Tetrachloroethane | 79-34-5 | 6.910 | 0.052 | 0.028 |
| 49 | 1,2,3-Trichloropropane | 96-18-4 | 7.011 | 0.039 | 0.021 |
| 50 | Isopropylbenzene | 98-82-8 | 7.086 | 0.037 | 0.017 |
| 51 | trans-1,4-Dichloro-2-butene | 110-57-6 | 7.131 | 0.103 | 0.054 |
| 52 | Bromobenzene | 108-86-1 | 7.176 | 0.048 | 0.022 |
| 53 | alpha-pinene | 7785-70-8 | 7.232 | 0.030 | 0.017 |
| 54 | 2-Chlorotoluene | 95-49-8 | 7.483 | 0.038 | 0.021 |
| 55 | Propylbenzene | 103-65-1 | 7.517 | 0.038 | 0.018 |
| 56 | 3-Ethylpyridine | 536-78-7 | 7.232 | 0.630 | 0.332 |
| 57 | 4-Chlorotoluene | 106-43-4 | 7.570 | 0.067 | 0.036 |
| 58 | 1,3,5-Trimethylbenzene | 108-67-8 | 7.746 | 0.047 | 0.023 |
| 59 | Pentachloroethane | 76-01-7 | 7.869 | 0.058 | 0.033 |
| 60 | Phenol | 108-95-2 | 7.899 | 0.364 | 0.171 |
| 61 | tert-Butylbenzene | 98-06-6 | 8.158 | 0.028 | 0.017 |
| 62 | 1,2,4-Trimethylbenzene | 95-63-6 | 8.169 | 0.038 | 0.018 |
| 63 | n-Decane | 124-18-5 | 8.244 | 0.034 | 0.023 |
| 64 | 1,4-Dichlorobenzene | 106-46-7 | 8.387 | 0.021 | 0.009 |
| 65 | sec-Butylbenzene | 135-98-8 | 8.484 | 0.024 | 0.015 |
| 66 | 1,3-Dichlorobenzene | 541-73-1 | 8.507 | 0.037 | 0.020 |
| 67 | 1,2,3-Trimethylbenzene | 87-61-6 | 8.286 | 0.190 | 0.113 |
| 68 | p-Isopropyltoluene | 99-87-6 | 8.747 | 0.025 | 0.015 |
| 69 | d-Limonene | 5989-27-5 | 8.833 | 0.084 | 0.052 |
| 70 | 1,2-Dichlorobenzene | 95-50-1 | 8.964 | 0.023 | 0.013 |
| 71 | Butylbenzene | 104-51-8 | 9.384 | 0.039 | 0.024 |
| 72 | Hexachloroethane | 67-72-1 | 9.733 | 0.047 | 0.029 |
| 73 | 1,2-Dibromo-3-chloropropane | 96-12-8 | 9.894 | 0.026 | 0.015 |
| 74 | Nitrobenzene | 98-95-3 | 9.976 | 0.143 | 0.073 |
| 75 | n-Undecane | 1120-21-4 | 10.145 | 0.032 | 0.024 |
| 76 | 1,2,4-Trichlorobenzene | 120-82-1 | 11.431 | 0.042 | 0.025 |
| 77 | Naphthalene | 91-20-3 | 11.532 | 0.052 | 0.025 |
| 78 | n-Dodecane | 112-40-3 | 11.674 | 0.041 | 0.032 |
| 79 | 1,2,3-Trichlorobenzene | 87-61-6 | 11.933 | 0.034 | 0.020 |

| # | Target VOCs | CAS | RT (min) | MDLs (ng) | MDL-3d (μg/m³) |
|---|---|---|---|---|---|
| 80 | Hexachlorobutadiene | 87-68-3 | 11.978 | 0.035 | 0.024 |
| 81 | n-Tridecane | 629-50-5 | 12.799 | 0.068 | 0.050 |
| 82 | Nicotine | 54-11-5 | 13.159 | 10.00 | 1.097 |
| 83 | n-Tetradecane | 629-59-4 | 13.549 | 0.057 | 0.044 |
| 84 | n-Pentadecane | 629-62-9 | 14.118 | 0.095 | 0.088 |
| 85 | Ethyl Alcohol | 64-17-5 | 1.681 | 0.032 | 0.042 |
| 86 | Isopropyl Alcohol | 67-63-0 | 1.759 | 0.031 | 0.047 |
| 87 | 2-Butoxyethanol | 111-76-2 | 6.750 | 0.311 | 0.256 |
| 88 | Benzaldehyde | 100-52-7 | 7.520 | 0.314 | 0.238 |
| 89 | beta-Pinene | 18172-67-3 | 7.810 | 0.407 | 0.399 |
| 90 | alpha-Terpinene | 99-86-5 | 8.490 | 0.137 | 0.130 |
| 91 | 2-Ethyl-1-hexanol | 104-76-7 | 8.670 | 1.415 | 1.216 |
| 92 | Benzyl alcohol | 100-51-6 | 8.780 | 0.306 | 0.212 |
| 93 | Salicylaldehyde | 90-02-8 | 9.000 | 0.278 | 0.202 |
| 94 | Terpinolene | 586-62-9 | 9.890 | 0.227 | 0.206 |
| 95 | Linalool | 78-70-6 | 10.050 | 0.265 | 0.237 |
| 96 | Camphor | 76-22-2 | 10.850 | 0.117 | 0.099 |
| 97 | Menthol | 89-78-1 | 11.250 | 0.090 | 0.077 |
| 98 | 1-Fluoronaphthalene | 321-38-0 | 11.440 | 0.324 | 0.228 |
| 99 | Terpineol | 98-55-5 | 11.510 | 0.297 | 0.244 |
| 100 | Methyl salicylate | 119-36-8 | 11.570 | 0.249 | 0.183 |
| 101 | 4-Allylanisole | 140-67-0 | 11.610 | 0.359 | 0.287 |
| 102 | Methyl-2-octynoate | 111-12-6 | 11.620 | 0.311 | 0.248 |
| 103 | Citronellol | 106-22-9 | 11.960 | 0.160 | 0.131 |
| 104 | Citronellol-Nerol-Geraniol | 5392-40-5 | 11.960 | 0.084 | 0.068 |
| 105 | (S)-(+)-Carvone | 2244-16-8 | 12.210 | 0.208 | 0.159 |
| 106 | trans-Cinnamaldehyde | 14371-10-9 | 12.490 | 0.310 | 0.206 |
| 107 | Anise alcohol | 105-13-5 | 12.600 | 0.309 | 0.196 |
| 108 | Hydroxycitronellal | 107-75-5 | 12.620 | 0.110 | 0.087 |
| 109 | Anethole | 104-46-1 | 12.640 | 0.140 | 0.105 |
| 110 | Safrole | 94-59-7 | 12.690 | 0.254 | 0.193 |
| 111 | Methyl 2-nonynoate | 111-80-8 | 12.750 | 0.310 | 0.246 |
| 112 | Cinnamyl alcohol | 104-54-1 | 12.820 | 0.290 | 0.194 |
| 113 | Dimethylbenzylcarbinyl acetate (DMBCA) | 151-05-3 | 12.940 | 0.135 | 0.109 |
| 114 | Eugenol | 97-53-0 | 13.240 | 0.149 | 0.108 |
| 115 | alpha-Damascone | 43052-87-5 | 13.390 | 0.150 | 0.123 |
| 116 | beta-Damascone(E) | 23726-91-2 | 13.450 | 0.026 | 0.021 |
| 117 | Damascenone (Rose Ketone-4) | 23696-85-7 | 13.510 | 0.101 | 0.083 |
| 118 | Vanillin | 121-33-5 | 13.540 | 0.309 | 0.187 |
| 119 | Methyl eugenol | 93-15-2 | 13.540 | 0.237 | 0.180 |

| # | Target VOCs | CAS | RT (min) | MDLs (ng) | MDL-3d (μg/m³) |
|---|---|---|---|---|---|
| 120 | beta-Caryophyllene | 87-44-5 | 13.730 | 0.282 | 0.238 |
| 121 | Coumarin | 91-64-5 | 13.820 | 0.262 | 0.142 |
| 122 | Isoegugenol | 5932-68-3 | 13.850 | 0.299 | 0.207 |
| 123 | alpha-Isomethylionone | 127-51-5 | 14.040 | 0.396 | 0.320 |
| 124 | Eugenyl acetate | 93-28-7 | 14.230 | 0.060 | 0.046 |
| 125 | Butylphenyl methylpropional | 80-54-6 | 14.270 | 0.070 | 0.055 |
| 126 | Amyl salicylate | 2050-08-0 | 14.490 | 0.273 | 0.200 |
| 127 | 3-Propylidene phthalide | 17369-59-4 | 14.510 | 0.182 | 0.123 |
| 128 | Isoeugenyl acetate | 93-29-8 | 14.640 | 0.311 | 0.229 |
| 129 | alpha-Amylcinnamaldehyde | 78605-96-6 | 14.800 | 0.145 | 0.110 |
| 130 | alpha-Santalol | 115-71-9 | 14.930 | 0.278 | 0.214 |
| 131 | alpha-Amylcinnamyl alcohol | 101-85-9 | 14.940 | 0.244 | 0.166 |
| 132 | Farnesol | 4602-84-0 | 15.050 | 0.313 | 0.256 |
| 133 | beta-Santalol | 77-42-9 | 15.100 | 0.222 | 0.166 |
| 134 | alpha-Hexylcinnamaldehyde | 101-86-0 | 15.180 | 0.311 | 0.231 |
| 135 | Benzyl Benzoate | 120-51-4 | 15.270 | 0.310 | 0.210 |
| 136 | Galaxolide(1+2) | 1222-05-5 | 15.590 | 1.538 | 1.196 |
| 137 | Benzyl salicylate | 118-58-1 | 15.640 | 0.265 | 0.184 |
| 138 | Hexadecanolactone/Dihydroambrettolide | 109-29-5 | 15.880 | 0.218 | 0.156 |
| 139 | Benzyl cinnamate | 103-41-3 | 16.320 | 0.315 | 0.195 |

**Table S3.** Spatial variation of VOC concentrations (µg/m³) in two hotel rooms.

| VOCs | Hotel 1 (Memphis) | | | Hotel 2 (Pittsburgh) | | |
|---|---|---|---|---|---|---|
| | Area 1 | Area 2 | Per Diff (%) | Bathroom | Bedroom | Per Diff (%) |
| **Alcohols** | **5,069** | **4,674** | **8** | **934** | **791** | **17** |
| 2-Butoxyethanol | 119 | 126 | 5 | 7.26 | 6.50 | 11 |
| 2-Ethyl-1-hexanol | 99.1 | 103 | 4 | 4.88 | 3.77 | 26 |
| Benzyl alcohol | 5.80 | 5.42 | 7 | 4.72 | 0.88 | 137 |
| Ethyl alcohol | 1,751 | 1,442 | 19 | 694 | 576 | 19 |
| Isopropyl alcohol | 3,094 | 2,998 | 3 | 223 | 203 | 9 |
| **Alkanes** | **269** | **266** | **1** | **7.18** | **6.95** | **3** |
| n-Decane | 1.33 | 1.48 | 10 | 0.59 | 0.32 | 59 |
| n-Dodecane | 2.49 | 2.49 | 0.2 | 1.44 | 1.36 | 6 |
| n-Nonane | 0.43 | 0.43 | 0.04 | 0.25 | 0.21 | 17 |
| n-Octane | 0.79 | 0.45 | 54 | 0.29 | 0.24 | 19 |
| n-Pentadecane | 69.7 | 69.2 | 1 | 0.46 | 0.87 | 60 |
| n-Tetradecane | 180 | 177 | 2 | 0.39 | 0.78 | 68 |
| n-Tridecane | 13.1 | 14.1 | 7 | 2.61 | 2.25 | 15 |
| n-Undecane | 0.83 | 0.88 | 6 | 1.15 | 0.92 | 22 |
| **Aromatics** | **37.0** | **37.2** | **0.5** | **4.73** | **4.16** | **13** |
| 1,2,3-Trimethylbenzene | 0.43 | 0.41 | 5 | 0.26 | 0.24 | 11 |
| 1,2,4-Trichlorobenzene | 0.05 | 0.04 | 23 | n.a. | n.a. | n.a. |
| 1,2,4-Trimethylbenzene | 1.10 | 1.12 | 2 | 0.26 | 0.24 | 11 |
| 1,3,5-Trimethylbenzene | 0.24 | 0.26 | 6 | 0.06 | 0.05 | 21 |
| 1,4-Dichlorobenzene | 11.2 | 11.4 | 2 | 0.04 | 0.03 | 10 |
| Benzene | 3.87 | 3.56 | 8 | 0.55 | 0.57 | 3 |
| Chlorobenzene | 0.07 | 0.06 | 13 | n.a. | n.a. | n.a. |
| Ethylbenzene | 0.95 | 0.96 | 1 | 0.29 | 0.25 | 14 |
| Isopropylbenzene | 0.13 | 0.12 | 5 | 0.05 | 0.04 | 18 |
| m,p-Xylene | 3.43 | 3.49 | 2 | 0.96 | 0.83 | 15 |
| Naphthalene | 4.57 | 4.59 | 0.5 | 0.12 | 0.13 | 5 |
| o-Xylene | 1.43 | 1.43 | 0.3 | 0.37 | 0.31 | 18 |
| p-Isopropyltoluene | 2.21 | 2.28 | 3 | 0.46 | 0.33 | 32 |
| Propylbenzene | 0.27 | 0.28 | 2 | 0.10 | 0.08 | 21 |
| Styrene | 1.86 | 1.89 | 2 | 0.27 | 0.24 | 14 |
| Toluene | 5.25 | 5.35 | 2 | 0.95 | 0.84 | 12 |
| **Carbonyl & Ketones** | **34.9** | **33.4** | **21** | **3.02** | **4.33** | **36** |
| alpha-Hexylcinnamaldehyde | 0.35 | 0.40 | 15 | 0.00 | 0.00 | n.a. |
| Benzaldehyde | 28.6 | 26.2 | 9 | 3.02 | 4.33 | 36 |
| Butylphenyl methylpropional | 0.42 | 0.47 | 11 | n.a. | n.a. | n.a. |
| Salicylaldehyde | 1.25 | 1.34 | 7 | n.a. | n.a. | n.a. |
| Damascenone (Rose Ketone-4) | 2.14 | 2.48 | 15 | n.a. | n.a. | n.a. |
| **Ethers & Esters** | **24.2** | **27.3** | **12** | **3.07** | **2.91** | **6** |

| VOCs | Hotel 1 (Memphis) | | | Hotel 2 (Pittsburgh) | | |
|---|---|---|---|---|---|---|
| | Area 1 | Area 2 | Per Diff (%) | Bathroom | Bedroom | Per Diff (%) |
| Amyl salicylate | 0.41 | 0.43 | 5 | n.a. | n.a. | n.a. |
| Benzyl Benzoate | 0.56 | 0.70 | 21 | n.a. | n.a. | n.a. |
| Ethyl Acetate | 8.15 | 9.56 | 16 | 1.28 | 1.16 | 10 |
| Methyl salicylate | 4.51 | 4.51 | 0.002 | 0.68 | 0.68 | 0.9 |
| 4-Allylanisole | 1.61 | 1.69 | 5 | n.a. | n.a. | n.a. |
| Anethole | 1.39 | 1.45 | 4 | n.a. | n.a. | n.a. |
| Galaxolide(1+2) | 0.52 | 0.66 | 24 | n.a. | n.a. | n.a. |
| Tetrahydrofuran | 7.10 | 8.29 | 15 | 1.10 | 1.06 | 4 |
| **Halocarbons** | **14.4** | **14.5** | **0.7** | **4.65** | **3.22** | **36** |
| 1,2-Dichloroethane | 0.35 | 0.33 | 6 | 0.05 | 0.05 | 6 |
| Carbontetrachloride | 0.90 | 0.92 | 2 | 0.47 | 0.42 | 12 |
| Chloroform | 12.6 | 12.7 | 0.9 | 4.02 | 2.65 | 41 |
| Tetrachloroethylene | 0.50 | 0.48 | 3 | 0.11 | 0.10 | 10 |
| **Terpenes** | **154** | **158** | **3** | **27.4** | **19.4** | **34** |
| alpha-Pinene | 11.9 | 12.0 | 1 | 0.59 | 0.42 | 33 |
| alpha-Terpinene | 1.49 | 1.55 | 4 | 3.31 | 2.44 | 30 |
| beta-Pinene | 8.17 | 8.14 | 0.4 | 1.01 | 0.80 | 23 |
| Citronellol-Nerol-Geraniol | 7.35 | 7.81 | 6 | n.a. | n.a. | n.a. |
| d-Limonene | 62.6 | 63.6 | 2 | 10.4 | 7.91 | 27 |
| Linalool | 39.1 | 40.9 | 5 | 4.08 | 2.25 | 58 |
| Menthol | 12.9 | 13.5 | 5 | 2.89 | 2.38 | 19 |
| Terpineol | 7.95 | 8.45 | 6 | 0.49 | 0.51 | 5 |
| Terpinolene | 2.38 | 2.06 | 14 | 4.60 | 2.70 | 52 |
| **ΣVOCs** | **5,601** | **5,208** | **7** | **984** | **832** | **17** |

**Table S4.** VOC concentrations (μg/m$^3$) measured by passive and active sampling methods in a hotel room.

| VOCs | Passive (3-day) | Active_Ave (20 min) | Day-1 Active (20 min) | Day-2 Active (20 min) | Day-3 Active (20 min) |
|---|---|---|---|---|---|
| **Alcohols** | **4,872** | **1,043** | **835** | **1,115** | **1,179** |
| 2-Butoxyethanol | 122 | 65.2 | 58.0 | 57.5 | 80.0 |
| 2-Ethyl-1-hexanol | 101 | 51.0 | 49.6 | 50.6 | 52.9 |
| Benzyl alcohol | 5.61 | 3.47 | 3.26 | 3.32 | 3.83 |
| Ethyl alcohol | 1,597 | 286 | 186 | 360 | 312 |
| Isopropyl alcohol | 3,046 | 637 | 538 | 644 | 729 |
| **Alkanes** | **267** | **319** | **304** | **318** | **336** |
| n-Decane | 1.40 | 1.53 | 1.48 | 1.59 | 1.54 |
| n-Dodecane | 2.49 | 3.28 | 3.19 | 3.42 | 3.24 |
| n-Nonane | 0.43 | 0.51 | 0.51 | 0.50 | 0.50 |
| n-Octane | 0.62 | 0.42 | 0.33 | 0.58 | 0.35 |
| n-Pentadecane | 69.5 | 99.1 | 94.5 | 96.3 | 106 |
| n-Tetradecane | 179 | 189 | 181 | 188 | 197 |
| n-Tridecane | 13.6 | 24.9 | 21.6 | 26.7 | 26.5 |
| n-Undecane | 0.85 | 0.87 | 0.89 | 0.91 | 0.82 |
| **Aromatics** | **37.1** | **40.4** | **37.8** | **41.2** | **42.1** |
| 1,2,3-Trimethylbenzene | 0.42 | 0.50 | 0.52 | 0.52 | 0.47 |
| 1,2,4-Trichlorobenzene | 0.05 | 0.03 | 0.02 | 0.03 | 0.03 |
| 1,2,4-Trimethylbenzene | 1.11 | 1.29 | 1.33 | 1.37 | 1.18 |
| 1,3,5-Trimethylbenzene | 0.25 | 0.28 | 0.29 | 0.29 | 0.25 |
| 1,4-Dichlorobenzene | 11.3 | 12.7 | 12.3 | 12.7 | 13.0 |
| Benzene | 3.71 | 2.82 | 1.91 | 3.01 | 3.52 |
| Chlorobenzene | 0.07 | 0.07 | 0.06 | 0.08 | 0.06 |
| Ethylbenzene | 0.95 | 1.05 | 0.94 | 1.06 | 1.15 |
| Isopropylbenzene | 0.13 | 0.13 | 0.13 | 0.14 | 0.12 |
| m,p-Xylene | 3.46 | 3.91 | 3.57 | 3.96 | 4.19 |
| Naphthalene | 4.58 | 5.76 | 5.64 | 5.83 | 5.80 |
| o-Xylene | 1.43 | 1.50 | 1.30 | 1.51 | 1.68 |
| p-Isopropyltoluene | 2.24 | 2.67 | 2.56 | 2.79 | 2.67 |
| Propylbenzene | 0.27 | 0.32 | 0.33 | 0.33 | 0.29 |
| Styrene | 1.87 | 2.03 | 1.88 | 2.08 | 2.13 |
| Toluene | 5.30 | 5.38 | 5.06 | 5.52 | 5.54 |
| **Carbonyls & Ketones** | **30.8** | **14.0** | **13.6** | **13.6** | **14.5** |
| alpha-Hexylcinnamaldehyde | 0.37 | 0.46 | 0.46 | 0.44 | 0.48 |
| Benzaldehyde | 27.4 | 10.6 | 10.3 | 10.5 | 11.0 |
| Butylphenyl methylpropional | 0.45 | 0.42 | 0.41 | 0.39 | 0.45 |
| Salicylaldehyde | 1.29 | 0.47 | 0.42 | 0.48 | 0.52 |
| Damascenone (Rose Ketone-4) | 2.31 | 1.99 | 2.03 | 1.79 | 2.14 |

| VOCs | Passive (3-day) | Active_Ave (20 min) | Day-1 Active (20 min) | Day-2 Active (20 min) | Day-3 Active (20 min) |
|---|---|---|---|---|---|
| **Ethers & Esters** | **25.8** | **18.5** | **16.2** | **20.8** | **18.5** |
| Amyl salicylate | 0.42 | 0.35 | 0.33 | 0.34 | 0.39 |
| Benzyl Benzoate | 0.63 | 0.46 | 0.44 | 0.44 | 0.50 |
| Ethyl Acetate | 8.86 | 7.88 | 6.68 | 9.37 | 7.60 |
| Methyl salicylate | 4.51 | 2.88 | 2.68 | 2.90 | 3.05 |
| 4-Allylanisole | 1.65 | 0.89 | 0.86 | 0.90 | 0.90 |
| Anethole | 1.42 | 0.84 | 0.81 | 0.86 | 0.86 |
| Galaxolide(1+2) | 0.59 | 0.77 | 0.75 | 0.70 | 0.85 |
| Tetrahydrofuran | 7.69 | 4.44 | 3.68 | 5.28 | 4.35 |
| **Halocarbons** | **14.4** | **7.83** | **7.11** | **8.79** | **7.59** |
| 1,2-Dichloroethane | 0.34 | 0.19 | 0.19 | 0.22 | 0.17 |
| Carbontetrachloride | 0.91 | 0.28 | 0.25 | 0.30 | 0.29 |
| Chloroform | 12.7 | 6.86 | 6.24 | 7.74 | 6.60 |
| Tetrachloroethylene | 0.49 | 0.49 | 0.43 | 0.53 | 0.52 |
| **Terpenes** | **156** | **128** | **124** | **135** | **123** |
| alpha-Pinene | 11.9 | 13.8 | 12.7 | 15.6 | 13.0 |
| alpha-Terpinene | 1.52 | 0.82 | 0.82 | 0.89 | 0.76 |
| beta-Pinene | 8.15 | 4.57 | 4.30 | 5.18 | 4.23 |
| Citronellol-Nerol-Geraniol | 7.58 | 5.16 | 3.80 | 5.79 | 5.88 |
| d-Limonene | 63.1 | 62.7 | 63.8 | 65.1 | 59.1 |
| Linalool | 40.0 | 25.1 | 24.0 | 26.9 | 24.3 |
| Menthol | 13.2 | 9.00 | 8.63 | 8.97 | 9.40 |
| Terpineol | 8.20 | 5.49 | 5.14 | 5.55 | 5.78 |
| Terpinolene | 2.22 | 1.06 | 1.19 | 1.15 | 0.83 |
| **ΣVOCs** | **5,404** | **1,570** | **1,338** | **1,652** | **1,721** |

**Table S5.** Potential Risk of Major VOC groups and selected predominant VOC species detected in hotel rooms, measured in μg/m$^3$.

| VOCs | Hotel 1 | Hotel 2 | Hotel 3 | Hotel 4 | Median | MRLs | HQ |
|---|---|---|---|---|---|---|---|
| **Alcohols** | **4,872** | **838** | **115** | **96** | **477** | | |
| Ethyl alcohol | 1,597 | 615 | n.a. | n.a. | 1,106 | n.a. | |
| Isopropyl alcohol | 3,046 | 210 | n.a. | n.a. | 1,628 | n.a. | |
| 2-Butoxyethanol | 122 | 6.75 | 52.9 | 3.71 | 29.8 | $2.9\times10^3$ | 0.01 |
| 2-Ethyl-1-hexanol | 101 | 4.14 | 59.0 | 84.0 | 71.5 | n.a. | |
| **Halocarbons** | **14.4** | **3.70** | **0.35** | **0.45** | **2.07** | | |
| Chloroform | 12.7 | 3.11 | 0.01* | 0.01* | 1.56 | 490 | 0.003 |
| **Aromatics** | **37.1** | **4.38** | **13.8** | **41.0** | **25.5** | | |
| 1,4-Dichlorobenzene | 11.3 | 0.03* | 0.24 | 0.22 | 0.23 | $1.2\times10^4$ | 0.00002 |

| VOCs | Hotel 1 | Hotel 2 | Hotel 3 | Hotel 4 | Median | MRLs | HQ |
|---|---|---|---|---|---|---|---|
| Benzene | 3.71 | 0.56 | 2.73 | 27.9 | 3.22 | 29 | 0.11 |
| Toluene | 5.30 | 0.88 | 2.32 | 5.08 | 3.70 | $7.5\times10^3$ | 0.0005 |
| Ethylbenzene | 0.95 | 0.27 | 0.65 | 0.71 | 0.68 | $2.2\times10^4$ | 0.00003 |
| Xylenes | 4.89 | 1.20 | 2.80 | 3.50 | 3.15 | $8.7\times10^3$ | 0.0004 |
| Styrene | 1.87 | 0.25 | 2.83 | 0.35 | 1.11 | $2.1\times10^4$ | 0.0001 |
| Naphthalene | 4.58 | 0.13 | 0.21 | 0.23 | 0.22 | n.a. | |
| **Alkanes** | **267** | **7.03** | **90.9** | **15.8** | **53.3** | | |
| n-Tetradecane | 179 | 0.65 | 6.05 | 4.86 | 5.46 | n.a. | |
| n-Pentadecane | 69.5 | 0.73 | 1.87 | 1.66 | 1.76 | n.a. | |
| **Terpenes** | **156** | **22.1** | **94.4** | **48.6** | **71.5** | | |
| d-Limonene | 63.1 | 8.75 | 57.5 | 17.7 | 37.6 | n.a. | |
| β-Pinene | 8.15 | 0.87 | 4.18 | 2.72 | 3.45 | n.a. | |
| α-Terpinene | 1.52 | 2.73 | 14.6 | 0.07 | 2.13 | n.a. | |
| Linalool | 40.0 | 2.86 | 0.12* | 7.60 | 5.23 | n.a. | |
| Menthol | 13.2 | 2.55 | 9.87 | 15.5 | 11.5 | n.a. | |
| Terpineol | 8.20 | 0.50 | 0.12* | 0.12* | 0.31 | n.a. | |
| **Ethers & Esters** | **25.8** | **3.96** | **7.47** | **4.07** | **5.77** | | |
| Ethyl Acetate | 8.86 | 1.20 | 6.33 | 2.94 | 4.64 | n.a. | |
| Methyl salicylate | 4.51 | 0.68 | 0.09* | 0.09* | 0.39 | n.a. | |
| Tetrahydrofuran | 7.69 | 1.08 | 0.04* | 0.04* | 0.56 | n.a. | |
| **Carbonyls** | **29.5** | **4.14** | **13.7** | **13.8** | **13.8** | | |
| Benzaldehyde | 27.4 | 3.89 | 13.5 | 13.5 | 13.5 | n.a. | |
| **ΣVOCs** | **5,404** | **882** | **339** | **217** | **610** | | **0.13** |

* - indicate values below the detection limit have been replaced with half of the method detection limit (MDL); MRLs - Minimal risk levels for hazardous substances by ATSDR; HQ – Hazard Quotient.
